# Kaempferol and Vitamin E Improve Production Performance by Linking the Gut–Uterus Axis Through the Reproductive Hormones and Microbiota of Late-Laying Hens

**DOI:** 10.3390/ani15010015

**Published:** 2024-12-25

**Authors:** Jing Zhang, Jie Zhang, Kangle Li, Xinyue Fu, Yanhui Liang, Minling Zhang, Shaolong Zhuang, Yuyun Gao

**Affiliations:** 1 Department of Animal Science, College of Animal Science, Fujian Agriculture and Forestry University, Fuzhou 350002, China; zj1532571033@163.com (J.Z.); likanglesoon@163.com (K.L.); 18282134081@163.com (X.F.); 13599873290@163.com (Y.L.); zml15819710478@163.com (M.Z.); gaoyuyun2000@163.com (Y.G.); 2 Fujian Hexing Ecological Agriculture Science and Technology Co., Ltd., Quanzhou 362801, China; zhuangshaolong2024@163.com

**Keywords:** late-laying hen, kaempferol, vitamin E, production performance, gut–uterus axis

## Abstract

This study evaluated the effects of kaempferol (KAE), and vitamin E (VE) on the performance, reproductive hormones, and the composition of the cecum and uterus microbiota in late-laying hens. A total of 192 49-week-old Jinghong No. 1 laying hens were randomly divided into four groups, with six replicates in each group and eight laying hens in each replicate, pre-reared for one week and formally tested for ten weeks. The CON group was fed basal diets, the VE group, the KAE group, and the KAE + VE group were fed a basal diet to which was added 0.2 g/kg VE, 0.4 g/kg KAE, and 0.2 g/kg VE + 0.4 g/kg KAE, respectively. The results are as follows. Compared to the CON group, the VE group, the KAE group, and the KAE + VE group significantly increased the egg production rate, average daily egg weight and significantly decreased the feed-to-egg ratio. The VE + KAE group significantly improved the Haugh unit. The VE group, the KAE group, and the KAE + VE group considerably enhanced the eggshell strength, eggshell relative weight, eggshell thickness, yolk color, and relative yolk weight. The serum E_2_ and LH levels of the KAE group and the KAE + VE group and the serum FSH levels of the KAE + VE group were significantly higher. In the ovary, the KAE group and the KAE + VE group’s *ESR1* gene expression levels were significantly higher, and the KAE + VE group’s *FSHR* gene expression levels were markedly higher. In the uterus, the KAE group and the KAE + VE group’s *ESR1* gene expression levels were dramatically higher, and the KAE + VE group’s *ESR2* and *FSHR* gene expression levels were significantly higher. 16S rRNA gene sequencing revealed a significant aggregation of cecum and uterus colonies in the Beta diversity PCoA. In the cecum, Firmicutes, Bacteroidetes, and WPS-2 were the dominant phylums. In the uterus, the Firmicutes, Proteobacteria, and Bacteroidetes were the dominant phylums. The KAE + VE group’s F/B was significantly higher at the phylum level than in the CON group and the VE group. In summary, the addition of VE and KAE to the diet can improve the production performance of late-laying hens, increase the content of reproductive hormones, and stabilize the cecal and uterus microbiota, which may be related to the hormone and microbiota linkage of the gut–uterus axis.

## Simple Summary

Long and intensive laying cycles, the insufficient secretion of reproductive hormones, and unfavorable changes in the microbiota of the organism will lead to poorer egg production and egg quality in the late stages of laying hens. Both kaempferol and vitamin E have a variety of biological benefits such as anti-inflammatory, antioxidant, and immunomodulatory effects. There is a lack of information on the effects of a combination of the two on the production performance of late-laying hens. The aim of this trial was to investigate the effects of kaempferol and vitamin E on the production performance, reproductive hormones, and cecal and uterus microbiota of late-laying hens. The results showed that kaempferol and vitamin E improved the performance of late-laying hens through the gut–uterus axis associated with reproductive hormones and microbiota. This provides a solution and theoretical technical support for solving the problems of the egg production rate and egg quality decline of late-laying hens.

## 1. Introduction

In layer rearing, once the peak yield begins to decline, gradually entering the late stage of the laying period, the production performance and reproductive performance of laying hens decline [1,2]. This is mainly due to the long laying cycle, the insufficient secretion of reproductive hormones, and changes in the composition of the intestinal and uterus microbiota that adversely affect egg quality [2,3]. This is consistent with the common-sense understanding that the aging of livestock and poultry will reduce the reproductive performance, and the reproductive performance of female animals is negatively correlated with an increase in age [1].

Kaempferol (KAE) is a naturally occurring phytoflavonoid. The rhizome of the ginger plant kaempferia spp. is the main source of KAE, which has the molecular formula C_15_H_10_O_6_ and is characterized by a phenyl ring and four hydroxyl substituents [4]. KAE is similar to estrogen in chemical structure and activity [5]. Previous studies confirmed that using fat-soluble compounds as carriers can improve the stability of antitoxins in vivo [6]. Lipid solubility is proportional to the bioavailability and biological activity of phytoflavonoids [6,7,8]. Vitamin E (VE) as a kind of odorless and tasteless yellowish fat-soluble vitamin is a green feed additive with various biological functions such as antibacterial properties, antimicrobial properties, anti-oxidative stress properties, immune regulation, and the enhancement of the reproductive ability [9,10,11]. Because it cannot be synthesized in the body and can only be supplied exogenously, it is usually supplemented in the form of alpha-tocopherol [12]. It has been confirmed by the multiphasic systematic analysis of the increasing structure and organization of phytoflavonoids (quercetin, rutin) and VE in the lipid system that the two can be coupled in in vivo experiments without antagonism or negative interaction [6].

In a study of a rat model of anovulation, researchers measured metabolism-related factors in the uterus and serum and found that the combination of phytoflavonoids (quercetin) and VE had an estradiol-like effect, which was considered an alternative to estrogen replacement therapy [13]. In animal production, phytoflavonoids and VE improve the egg production, egg quality, and cecal microbiota in late-laying hens. Both have been confirmed to promote yolk precursor synthesis and follicular development in senescent species through the liver–blood–ovarian signaling axis [1]. Some studies found that phytoflavonoids and VE promoted the growth performance of broilers under high-temperature conditions. The results showed that the combined application of plant flavonoids and VE had a positive effect on energy and nutrient metabolism in livestock and poultry [14,15].

At present, the research on kaempferol is mainly focused on medicine. The application of KAE alone and the combined use of KAE + VE in layer production is rarely reported. This study investigated the effects of KAE and VE alone and in combination on the production performance, reproductive hormones and receptors, and cecum and uterus microbiota of late-laying hens; it aims to provide theoretical and technical support for solving the problem of the deterioration in production performance in the late-laying stage.

## 2. Materials and Methods

### 2.1. Moral Statement

This study was approved by the Fujian Agriculture and Forestry University Animal Care and Use Committee (Approval ID: PZCASFAFU23012).

### 2.2. Birds, Experimental Design, and Management

A total of 192 Jinghong No. 1 late-laying hens were obtained from Beijing Hua du Yu kou Poultry Industry Co., Ltd., (Beijing, China). They were fed a basal diet for 1 week (49 weeks) followed by a formal trial for 10 weeks (50–60 weeks). The experiment was conducted at the same facility. The hens were randomly assigned to 4 treatment groups consisting of 6 replicates of 8 birds each. They were fed corn and soybean meal-type diets. The lighting system was controlled (16 h of light per day) and optimal ventilation was maintained throughout the experimental period.

The CON group was fed a basal diet, the VE group was supplemented with 0.2 g/kg VE in the basal diet, the KAE group was supplemented with 0.4 g/kg KAE in the basal diet, and the KAE + VE group was supplemented with 0.2 g/kg VE and 0.4 g/kg KAE in the basal diet. The additional amount of KAE was based on the test results of Amevor et al. [1,16], and the additional amount of VE was based on the results of the study of Jafari et al. [13,17]. Table 1 shows the feed composition and nutrient levels for the experimental ration. KAE (98%, Sophora horn extract) was supplied by Shaanxi Fuerbang Biotechnology Co., Ltd., Xi’an, China. VE (DL-α-Tocopherol) was supplied by BASF (Shanghai, China) Vitamins Co., Ltd.

### 2.3. Sample Collection

The daily data were summarized in the unit of repetition, and the daily laying performance indicators were calculated according to the Measurement Indicators and Statistical Methods of Poultry Production Performance (NY/T823-2004) [19], which included the egg laying rate, daily egg weight, average daily feed intake, feed-to-egg ratio (F/E), and qualified egg rate. At the end of the feeding period (60 weeks), 48 late-laying hens were randomly selected, with 2 chickens in each replicate, and 10 mL of blood was collected from the wing vein; the blood samples were placed at room temperature for 1 h, centrifuged at 3000 r/min for 15 min, and the serum was aspirated and stored at −20 °C for spare use. Then, these late-laying hens were slaughtered, sampled, and analyzed. The ovaries were quickly separated, the contents of the cecum were separated, the reproductive tract was separated, the mucous of the uterus was scraped out, and the whole uterus was frozen in a freezing tube, frozen in liquid nitrogen, and stored at −80 °C.

### 2.4. Determination of Egg Quality Parameters

At the end of the 5th and 10th weeks of the experiment, two eggs were randomly taken from each replicate (a total of 96 eggs) for egg quality determination. Each egg collected was measured and evaluated for all quality parameters within 3 h. The Haugh unit and yolk color were determined according to a multifunctional egg quality tester (Robotmotion Co., Ltd., Takanawa Minato-ku, Tokyo, Japan). The eggshell thickness was determined by measuring the average thickness of the eggshell at three positions, the tip, blunt end, and middle, and the longitudinal diameter and transverse longitude of the eggs were measured with a vernier caliper. The eggshell strength was determined using an eggshell strength tester (Robotmotion Co., Ltd., Takanawa Minato-ku, Tokyo, Japan).

### 2.5. Serum Reproductive Hormone Determination

Serum levels of estradiol (E_2_), Follicle-Stimulating Hormone (FSH), Luteinizing Hormone (LH), and progesterone (PROG) were determined using commercially available specific enzyme-linked immunosorbent assay (ELISA) kits following the protocols provided by the manufacturer (Shanghai Enzyme-linked Biotechnology Co., Ltd., Shanghai, China).

### 2.6. RNA Extraction and Quantitative PCR

Total RNA was extracted from the ovary and uterus using TRIzol reagent (Tiangen, Beijing, China) according to the instructions, and the concentration and purity were determined by the A260/280 absorbance ratio using an ultra-micro spectrophotometer (YEASEN, Shanghai, China). First-strand cDNA was synthesized using the Hiffair Trademark^®^ III 1st Strand cDNA Synthesis SuperMix for qPCR kit (YEASEN, Shanghai, China) according to the manufacturer’s instructions. q-PCR was performed using a real-time fluorescence quantitative PCR instrument (Bio-Rad CFX96, Shanghai, China), and each reaction system was 20 μL, containing 10 μL of SYBR^®^ Premix EX TaqTM, 1 μL of forward primer, 1 μL of reverse primer, 1 μL of a cDNA template, and 7 μL of ddH_2_O. GAPDH was used as an endogenous control to normalize the gene expression, and three replicates of each sample were tested for the relative gene expression. Fold changes in expression were quantified using the 2^−ΔΔCt^ method, where ΔCt = the Ct target gene—the Ct housekeeping gene and ΔΔCt = ΔCt − the ΔCt reference gene [20]. Primer 5 software was utilized to design gene-specific primers for q-PCR analysis based on the coding sequences of the target genes (Table 2).

### 2.7. 16S rRNA Sequencing to Analyze the Microbiota Structure of Cecum Contents and Uterus Mucus

After extracting the genomic DNA from the samples, the purity and concentration of the DNA were detected using 1% agarose gel electrophoresis, and an appropriate amount of the sample DNA was put into a centrifuge tube and diluted in sterile water to 1 ng/μL. The diluted genomic DNA was used as a template, and according to the selection of sequencing regions, specific primers with barcode were used to amplify the 16S rRNA V3-V4 region. The library was constructed using a TruSeq^®^ DNA PCR-Free Sample Preparation Kit (Illumina, Inc., San Diego, CA, USA), and the constructed library was quantified using Qubit (Thermo Fisher Scientific, Waltham, MA, USA) and Q-PCR; after the library was quantified, it was sequenced on the IlluminaHiseq2500 platform using the Illumina sequencing platform double-end sequencing method (Paired-End). After the PE reads obtained from Illumina sequencing were split into samples, the double-end reads were first quality-controlled and filtered according to the sequencing quality, and at the same time spliced according to the overlapping relationship between the double-end reads to obtain the optimized data after quality-controlled splicing. The optimized data were then processed using sequence noise reduction methods (DADA2/Deblur) to obtain the ASV (Amplicon Sequence Variant) representative sequence and abundance information. Based on the ASV representative sequence and abundance information, a series of statistical or visualization analyses can be performed, such as species taxonomic analysis and community diversity analysis.

### 2.8. Statistical Analysis

After the experimental data were organized by Excel 2020, SPSS 26.0 statistical software was used to perform a one-way ANOVA and Duncan’s multiple comparison test and GraphPad Prism (8.0) was used for plotting, and all the experimental data were expressed as means ± the standard deviation (means ± SD). *p* < 0.01 was highly significant, 0.01 ≤ *p* < 0.05 was significant, and *p* ≥ 0.05 was not significant.

## 3. Results

### 3.1. Production Performance

The effects of adding VE, KAE, and KAE + VE on the production performance of late-laying hens are shown in Table 3. The results showed, compared with the CON group, that the KAE group, the VE group, and the KAE + VE group increased the egg laying rate (*p* < 0.01), and the KAE + VE group was higher in this respect than the VE group (*p* < 0.01), but the rates between the KAE group and the VE group did not differ (*p* > 0.05). The average daily egg weight of the other three groups was higher than that of the CON group (*p* < 0.05). The feed-to-egg ratio of the CON group was much higher than in the other three groups (*p* < 0.01), but the differences in the daily feed intake and qualified egg rate were not significant among the groups (*p* > 0.05).

### 3.2. Egg Quality

The effects of adding VE, KAE, and KAE + VE on the egg quality of late-laying hens are shown in Table 4. The results showed that the KAE + VE group increased the Haugh unit compared to the CON group (*p* < 0.05). The VE group, the KAE group, and the KAE + VE group improved the eggshell strength, relative eggshell weight, and eggshell thickness compared to the CON group (*p* < 0.05). In addition, the results in Table 4 show that the yolk color and relative yolk weight were higher in the VE group, the KAE group, and the KAE + VE group compared to the CON group (*p* < 0.01). However, for the egg shape index, there was no statistical difference between the groups (*p* > 0.05).

### 3.3. Serum Reproductive Hormones

**Figure 1:**
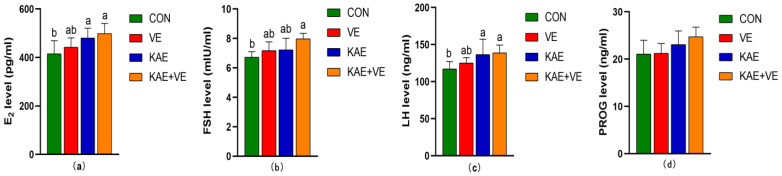
Effects of VE and KAE on serum hormone levels of late-laying hens. (**a**) E_2_, (**b**) FSH, (**c**) PROG, (**d**) LH. Different lowercase letter superscripts mean notable differences (*p* < 0.05). Values with the same or no letters mean no significant difference (*p* ≥ 0.05). Data are presented as the mean ± SD.

The effects of adding VE, KAE, and KAE + VE on the serum hormone levels of late-laying hens are shown in Figure 1. The results showed that, compared with the CON group, the serum E_2_ and LH levels in the KAE group and the KAE + VE group were increased (*p* < 0.05, Figure 1a,c). Serum FSH levels were higher in the KAE + VE group compared to the CON group (*p* < 0.05, Figure 1b), whereas the difference between the VE group and KAE group was not statistically significant (*p* > 0.05, Figure 1b); serum PROG levels did not show differences among the groups (*p* > 0.05, Figure 1d).

### 3.4. Reproductive Hormone Receptor Gene Expression Levels

**Figure 2:**
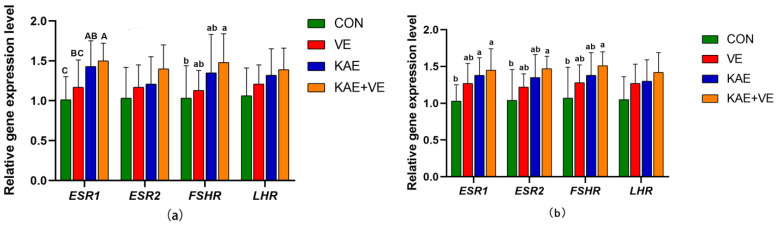
Effects of VE and KAE on gene expression levels of reproductive hormone receptors in ovary and uterus of late-laying hens. (**a**) Ovary, (**b**) Uterus. Different lowercase letter superscripts mean notable differences (*p* < 0.05), while different capital letters show significant differences (*p* < 0.01). Values with the same or no letters mean no significant difference (*p* ≥ 0.05). Data are presented as the mean ± SD.

The effects of adding VE, KAE, and KAE + VE on the gene expression levels of reproductive hormone receptors in the ovary and uterus of late-laying hens are shown in Figure 2. Figure 2a summarizes the results of hormone receptor gene expression in the ovary. The results showed that *ESR1* gene expression was higher in the ovary of the KAE group and KAE + VE group compared to the CON group (*p* < 0.01) and also exhibited an elevation in the ovary of the KAE + VE group compared to the VE group (*p* < 0.01). *FSHR* gene expression was higher in the ovary of the KAE + VE group compared to the CON group (*p* < 0.05).

The gene expression levels of reproductive hormone receptors in the uterus were similar to that of the ovary. Figure 2b summarizes the results of hormone receptor gene expression in the uterus. *ESR1* gene expression was higher in the uterus of the KAE group and KAE + VE group, compared to the CON group (*p* < 0.05), whereas the differences between the VE group, the KAE group, and the KAE + VE group were not statistically significant (*p* > 0.05). Compared with the CON group, the uterus of the KAE + VE group had higher *ESR2* and *FSHR* gene expression levels (*p* < 0.05), and the differences between the VE group and the KAE group were not statistically significant (*p* > 0.05).

### 3.5. Cecum Microbiota

**Figure 3:**
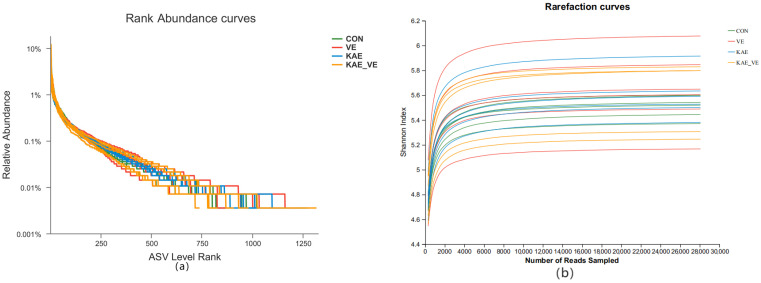

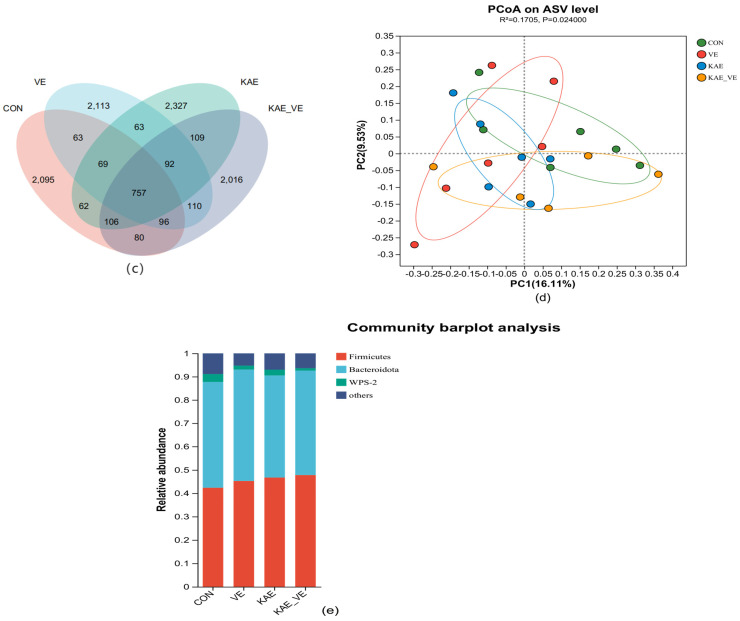
Effects of VE and KAE on cecal microbiota in late-laying hens. (**a**) Rank Abundance curve. (**b**) Rarefaction curve. (**c**) Venn diagram. (**d**) Principal coordinate analysis (PCoA) based on Bray–Curtis distance. (**e**) Relative abundance in composition at phylum level.

The bacterial 16S rRNA V3 + V4 region was sequenced to analyze the microbiota of cecal contents in different groups. The DADA2 method was used for sequence denoising. After the optimized sequences were obtained, the ASV level of the strains was obtained using classify-sklearn (Naive Bayes) for analysis. Rank Abundance curves visualized the abundance and homogeneity of species in a sample. As can be seen from the Rank Abundance curves (Figure 3a), the curve spans a greater distance on the horizontal axis, indicating that the abundance of species is higher in each sample. In the vertical direction, the smoothness of the curves is similar across the samples, indicating similar homogeneity across the samples. The Rarefaction curve can be used to compare the species richness of the different samples sequenced and determine whether the number of samples sequenced is sufficient. From the Rarefaction curve (Figure 3b), it can be seen that with an increase in the number of samples taken, the Shannon index tends to smooth out gradually, indicating that the number of sequenced samples is sufficiently large, which can concur with the information of the majority of microbial species in the samples, and the sequencing results are reasonable. The Venn diagram (Figure 3c) reflected a total of 757 species in each group, and there were 2095, 2113, 2327, and 2016 ASVs for species specific to the CON group, the VE group, the KAE group, and the KAE + VE group, respectively. Alpha-related indexes were counted for each group (Table 5), and it was found that the addition of VE, KAE, and KAE + VE to the diet did not affect the Alpha diversity of the cecum microbiota (ACE, Chao1, Sobs, and Shannon indices) (*p* > 0.05). As shown by the Beta diversity analysis in the PCoA (Figure 3d), there was a clustering of cecum microorganisms in each group (*p* < 0.05). The relative abundance in the cecum content microbiota of late-laying hens is presented at the phylum level (Figure 3e). At the phylum level, the cecum microbiota of 60-week-old laying hens was composed of Firmicutes, Bacteroidetes, and WPS-2, and these were the dominant phylums of the cecum microbiota. At the phylum level, Firmicutes and Bacteroides as well as their ratio showed little change (*p* > 0.05, Table 6).

### 3.6. Uterus Microbiota

**Figure 4:**
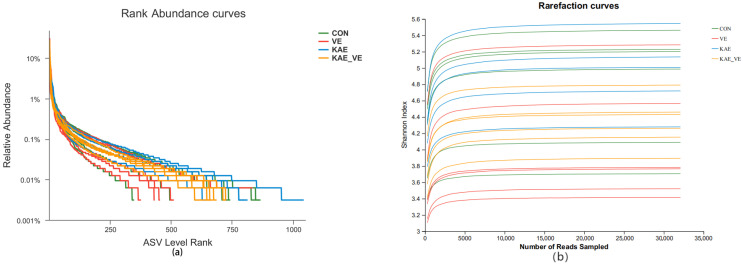

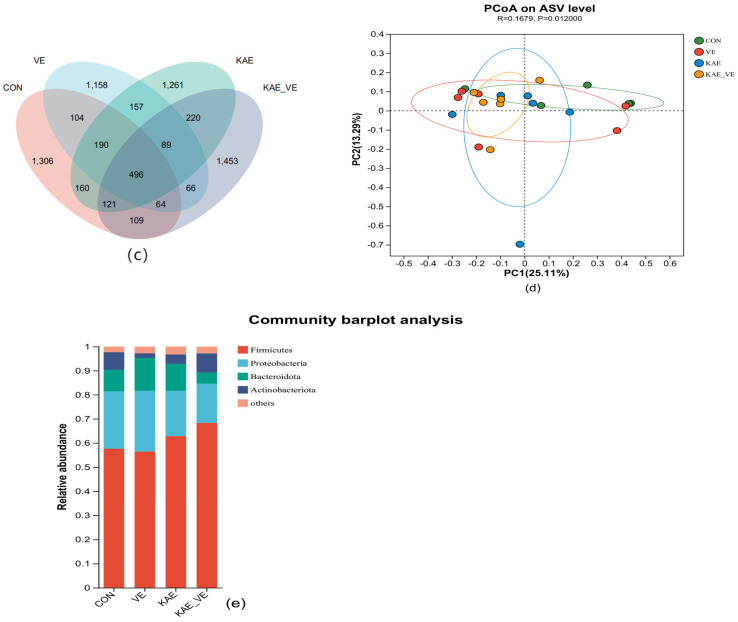
Effects of VE and KAE on uterus microbiota in late-laying hens. (**a**), Rank Abundance curve. (**b**) Rarefaction curve. (**c**) Venn diagram. (**d**) Principal coordinate analysis (PCoA) based on Bray−Curtis distance. (**e**) Relative abundance in composition at phylum level.

The same method was used to analyze the mucus microbiota of the different groups of uterus sites. Rank Abundance curves could visualize the abundance and homogeneity of the species in the samples. As can be seen from the Rank Abundance curves (Figure 4a), the curves have a larger span on the horizontal axis, indicating a higher abundance of the species in the individual samples; in the vertical direction, the curves of the samples have a comparable degree of smoothness, indicating that there is no significant difference in the samples. In the vertical direction, the smoothness of the curves of each sample is comparable, indicating that the distribution of species is not uniform. From the Rarefaction curve (Figure 4b), it can be seen that the Shannon index tends to be smoother with an increase in the number of samples taken, indicating that the sequencing quantity is large enough to reflect the information of the majority of microbial species in the samples and that the sequencing results are reasonable. The Venn diagram (Figure 4c) reflects that there are a total of 496 species in each group, and the species unique to the CON group, the VE group, the KAE group, and the KAE + VE group are 1306, 1158, 1261, and 1453 ASVs, respectively. Alpha-related indices were counted for each group (Table 7). Differences in the Alpha diversity of the microbiota of the uterus of the six groups were not significant (ACE, Chao1, Sobs, and Shannon indices). As shown by the Beta diversity analysis in the PCoA (Figure 4d), there was a clustering of the uterus microbiota in each group (*p* < 0.05). The relative abundance in the microbiota in the uterus of late-laying hens is presented at the phylum level (Figure 4e). At the phylum level, the microbiota in the uterus of 60-week-old laying hens were mainly composed of Firmicutes, Proteobacteria, and Bacteroidota, and these were the dominant phylums of the uterus. Compared with the CON group and the VE group, the F/B value at the phylum level in the KAE + VE group was increased (*p* < 0.05, Table 8).

## 4. Discussion

### 4.1. Effects of KAE and VE on Egg Laying Performance and Egg Quality in Late-Laying Hens

Studies have shown that physiological aging in laying hens lead to a decrease in egg production and poor egg quality [4]. As a typical plant phytoflavonoid, KAE has estrogen-like functions in animals and has the effect of promoting production [21]. VE can promote the release of vitelline from the liver, improve egg production rates, and improve egg quality [22].

It has been found that genistein significantly prolongs the laying cycle and improves the laying rate of laying hens [23]. Quercetin and daidzein also significantly increased egg production and the egg weight, and reduced the feed-to-egg ratio in laying hens [24,25]. In addition, VE significantly improved egg production performance in older hens [22]. Recent studies have found that the combination of quercetin + VE has better results for the performance of older hens compared to the use of either alone [15]. The results of this study were consistent with the above studies, and the addition of KAE, VE, and KAE + VE to the diet significantly increased the egg laying rate and average daily egg weight, while significantly reducing the feed-to-egg ratio. Compared with the addition of VE and KAE, the addition of KAE + VE showed a stronger pro-production capacity, which indicated that the addition of KAE + VE led to stronger estrogen-like activity and egg yolk synthesis abilities. In addition, dietary supplementation with VE, KAE, and KAE + VE had no significant effect on the feed intake and good egg rate, consistent with previous reports [1,26].

The larger the Haugh unit, the better the viscosity of the egg white [24]. It has been found that the combination of quercetin + VE significantly increases the protein quality in eggs [15]. Consistent with the results of this study, the addition of KAE + VE to the diet significantly increased the Haugh unit of eggs. However, the addition of VE and KAE alone did not significantly improve the Haugh unit, which may have been due to the influence of factors such as the age and breed of laying hens used or the type and dosage of phytoflavonoids used, which led to different conclusions.

Egg yolk is an important nutrient in eggs, rich in fat-soluble vitamins, unsaturated fatty acids, phosphorus, iron, and other nutrients. The color of egg yolk is determined by the fat-soluble pigments deposited into the egg yolk, which is also one of the important factors affecting egg quality [1]. VE has been found to improve the egg yolk color by promoting the release of lutein [27,28]. Phytoflavonoids + VE can improve the egg yolk color through the deposition of carotenoids and lutein [15]. This is consistent with the results of this study, as the addition of VE, KAE, and KAE + VE to the diet significantly improved the egg yolk color and relative yolk weight, and the addition of KAE + VE had the best effect. This suggests that the addition of VE, KAE, and KAE + VE to the diet can positively improve the quality of egg yolk by increasing the level of reproductive hormones and promoting the synthesis of egg yolk precursors [29].

Poor eggshell quality is a prominent problem in the later stages of egg laying in late-laying hens, including a decrease in the eggshell strength, eggshell thickness, and relative eggshell weight [30]. Phytoflavonoids have been reported to improve eggshell thickness by regulating calcium metabolism through estrogen-like effects [24]. Studies have found that mulberry leaf flavonoids improve eggshell quality by increasing the antioxidant capacity and calcium deposition of the uterine shell glands [31]. The amount of VE added was positively correlated with eggshell thickness and strength [32,33]. Phytoflavonoids + VE actively improve calcium metabolism during aging through hormonal pathways and improve eggshell quality [34]. This study also found that the addition of VE, KAE, and KAE + VE to the diet significantly improved eggshell quality, such as the eggshell strength, eggshell thickness, and relative eggshell weight, and adding KAE + VE had the best effect.

### 4.2. Effects of KAE and VE on the Expression of Reproductive Hormones and Receptors in Late-Laying Hens

Serum hormone levels are considered sensitive indicators of follicular development as well as egg-laying performance [35]. E_2_ secreted by the ovaries accelerates the proliferation of follicular granulosa cells. Insufficient E_2_ secretion can exacerbate follicular atresia and reduce egg production performance in poultry [36]. The hypothalamic–pituitary–gonadal axis regulates ovarian function by stimulating the pituitary gland to secrete gonadotropins, stimulates steroid synthesis in the gonads, and regulates the growth and development of follicles. FSH and LH can promote the maturation of follicular and granular layers and promote the secretion of PROG and E_2_ by follicular sheath cells and granulosa cells [37]. Many studies have shown that the biological effects of reproductive hormones such as E_2_, FSH, and LH on target cells are triggered by their receptors [38,39]. Follicular development is closely related to the development of reproductive organs and the gene expression of hormone receptors such as *FSHR* and *LHR*. When E_2_ and *ER* are combined, the expression of *LHR* in the ovaries can be upregulated and ovulation can be induced [40]. After FSH binds to *FSHR*, it increases aromatase levels in granulosa cells, stimulates the proliferation and differentiation of intracellular cAMP, and regulates ovarian and follicular growth [41].

KAE has a similar molecular structure to E_2_ and activates estrogen-associated gene expression [5]. VE promotes the transport and deposition of yolk precursors to oocytes [17]. Studies have shown that the combination of phytoflavonoids + VE has a strong estrogenic effect and promotes the secretion of reproductive hormones and binding to hormone receptors [13]. Phytoflavonoids + VE upregulated the levels of E_2_, FSH, and LH and their receptors in the ovaries of aged laying hens [16]. This study showed that the addition of KAE and KAE + VE to the diet increased the serum E_2_ and LH levels, and the addition of KAE + VE increased the FSH level. In the ovary, KAE and KAE + VE increased the expression of the *ESR1* gene, and KAE + VE increased the expression of the *FSHR* gene. In the uterus, KAE and KAE + VE increased the expression of *ESR1* genes, and KAE + VE increased the expression of *ESR1* and *FSHR* genes. This illustrates the powerful estrogen-like effects of KAE + VE added to the body, revealing the potential of the two to be used in combination to improve the performance of late-laying hens.

### 4.3. Effects of KAE and VE on Cecal Microbiota in Late-Laying Hens

The gut microbiota is a potential regulator of production performance and gut health [13]. Studies have shown that phytoflavonoids such as genistein, bamboo leaf flavonoids, and quercetin have positive effects in regulating aging intestinal homeostasis, restoring the intestinal microbiota activity, and increasing the abundance of beneficial bacteria [42,43]. VE maintains gut health by improving the composition of the cecal microbiota in laying hens [44]. The latest studies have shown that the addition of quercetin, VE, and quercetin + VE to the diet can improve the Beta diversity of the cecal microbiota [45], which is consistent with the results of this study, and there was significant clustering of the cecal microbiota in each group in the Beta diversity analysis, indicating that the addition of VE, KAE, and KAE + VE to the diet can optimize the cecal microbiota of late-laying hens. However, there was no significant difference in the Alpha diversity of the cecal microbiota between the groups, which may be related to the age of laying hens and the amount of phytoflavonoid additives.

Firmicutes can ferment indigestible carbohydrates in the diet and convert them into short-chain fatty acids that provide energy for the gut [46]. Bacteroides utilize complex polysaccharides to produce propionic acid and butyric acid, which provide nutrients and energy to the host gut [47]. WPS-2 is a class of bacteria newly discovered in recent years, which contains a variety of bacteria with metabolic capabilities. These bacteria are organoheterotrophic and amino acids and nucleotides are considered the substrates of choice, while other substrates include thiosulfates, formates, and possibly some oligopeptides or oligosaccharides [48]. This study found that the dominant phylums in late-laying hens were Firmicutes, Bacteroidetes, and WPS-2 in the late laying stage. Consistent with earlier reports, the first and second phylums in the cecum of laying hens were Firmicutes and Bacteroidetes [48,49]. WPS-2 became the third dominant phylum, which is speculated to be related to the increase in thiosulfate substrates and the biosynthesis of pigments such as carotenoids.

In this study, the addition of KAE and VE established a relatively stable intestinal microbiota dominated by Firmicutes, Bacteroidetes, and WPS-2 by altering the cecal microbiota structure, thus promoting the decomposition of indigestible carbohydrates and polysaccharides by the microbiota and the further fermenting of short-chain fatty acids, which play an active role in feed digestion and absorption. This may be one of the reasons for the performance changes in late-laying hens.

### 4.4. Effects of KAE and VE on the Uterus Microbiota in Late-Laying Hens

The reproductive tract microbiota of laying hens has a greater effect on egg laying performance than the digestive tract microbiota, and the uterus microbiota is closely related to egg quality [48,49]. In this study, it was found that there was a significant clustering of the uterus microbiota in each group in the Beta diversity analysis of the uterus microbiota, which indicated that the composition of the uterus microbiota was significantly affected by the diets supplemented with VE, KAE, and KAE + VE.

It has been reported that Firmicutes is the dominant bacterial taxon on the surface of eggshells, and the uterus microbiota of laying hens is also dominated by Firmicutes. This suggests that vertical microbiota transmission is one of the keys to egg safety [50]. Studies have shown that Proteobacteria is the phylum with the highest content in the internal contents of eggs. This coincides with high levels of Proteobacteria in the oviducts of laying hens [51]. It has been found that trimethylamine, which is not metabolized, is deposited in egg yolks and produces an unpleasant fishy smell, while Firmicutes, Proteobacteria, and Bacteroides regulate trimethylamine metabolism [52]. In addition, the dominant phylums of the uterus of laying hens are Firmicutes, Proteobacteria, and Bacteroides [52,53]. Consistent with the results of this study, at the phylum level, Firmicutes, Proteobacteria, and Bacteroidetes were dominant. In addition, this study also found that the addition of KAE + VE to the diet significantly increased the F/B value. The F/B ratio is believed to have an important relationship with the composition of the body’s microbiota [54,55], and a high proportion of F/B is highly correlated with material energy absorption, metabolism, and weight stability. It is speculated that a high proportion of F/B has a potential positive regulatory effect on the sustained maintenance of high-intensity egg production in late-laying hens.

### 4.5. Effects of KAE and VE on the Gut–Uterus Axis in Late-Laying Hens

Anatomically, the cloaca is the end of the reproductive and digestive tracts of poultry, suggesting that the intestinal and fallopian tube microbiota may be connected through the cloaca [56]. In medicine, it has also been reported that bacteria are transmitted from the gut to the uterus through the bloodstream, and the uterus microbiota is closely related to fertility and uterus-related diseases, suggesting that the gut–uterus axis regulates the uterus microbiota and plays a key role in female animal fertility [57,58]. Studies have also confirmed that the composition of the intestinal microbiota and the reproductive microbiota overlap greatly, and the intestinal tract and the reproductive tract communicate in multiple directions through the microbiota and the metabolites produced by the microbiota [59]. In addition, the intestinal microbiota affects the production capacity of female animals by regulating the secretion of reproductive hormones binding to their receptors and regulating the reproductive tract ecosystem, which shows the importance of optimizing the microbiota [60,61]. These studies have confirmed the existence of the gut–uterus axis and its important role in maintaining the production and development of female animals.

The microbiota affects the body’s hormone levels, and studies have shown that E_2_ and the microbiota are important mediators of the gut–uterus axis [62]. E_2_ is metabolized and regulated by β-glucuronidase. This enzyme breaks down estrogen and binds it to estrogen receptors. This suggests that the gut microbiota regulates estrogen levels by participating in the secretion of β-glucuronidase [63]. In addition, the intestinal microbiota can affect endogenous estrogen metabolism, produce or alter reproductive hormones such as FSH, and indirectly affect physiological activities such as follicular development and ovulation [64,65]. In this study, we found that the addition of KAE and VE to the diet had similar effects on the composition and structure of the cecal microbiota and uterus microbiota. In the β diversity analysis of the microbiota, the cecal and uterus microbiota had similar significant clusters. In addition, both VE and KAE could increase the level of E_2_ and the expression level of receptor genes, and the addition of KAE + VE had the best effect.

## 5. Conclusions

The addition of KAE and VE to the diet can affect the gut–uterus axis by increasing the level of reproductive hormones, enhancing the expression of ovarian and uterus hormone receptors, and improving the composition and structure of the cecum and uterus microbiota, thereby improving the production performance of late-laying hens. The combination of KAE + VE works best. This provides reliable reference data for the application of phytoflavonoids and vitamins in improving the production of late-laying hens and is a way to improve the performance degradation in the late production stage of egg production.

## Figures and Tables

**Table 1:** Composition and nutrient levels of basal diets (air-dried basis), %. The nutrition level refers to the Chicken Feeding Standard (NY/T 33-2004) [18].

Ingredients (%)	Content
Corn	62.00
Soybean meal	26.00
CaHPO_4_	1.00
Limestone	8.00
Premix ^1^	3.00
Total	100.00
Nutrient composition ^2^ (%)	
Metabolic energy, ME (MJ/kg)	11.20
Crude protein, CP	16.80
Lysine, Lys	0.78
Methionine + Cysteine, Met + Cys	0.66
Calcium, Ca	3.70
Available phosphorus, AP	0.26

^1^ The premix provides, per kg of feed, vitamin A: 8000 IU; vitamin D3: 3500 IU; vitamin E: 25 mg; vitamin K3: 2.5 mg; vitamin B1: 4.0 mg; vitamin B2: 6.0 mg; vitamin B6: 5.4 mg; vitamin B12: 24.0 μg; niacin: 35.0 mg; pantothenic acid: 15 mg; folic acid: 0.9 mg; biotin: 150 μg; choline chloride: 500 mg; iron: 65 mg; copper: 6.8 mg; manganese: 80 mg; zinc: 75 mg; iodine: 1.0 mg; selenium: 0.3 mg. ^2^ Nutrient contents were calculated values.

**Table 2:** Sequences of real-time PCR primers.

Target Gene	Primer Sequence (5′ → 3′)	Accession No.	Size (Bp)
GAPDH	F: AGTGAAGGCTGCTGCTGATGG R: TCAAAGGTGGAGGAATGGCTGTC	NM_204305.2	105
ESR1	F: GTTCCGCTCTACGACCTCTTACTG R: AGTTGGTTTCGGTTCTCCTCTTCC	NM_205183.2	104
ESR2	F: CCAGCTACCAATCAATGCACGATAG R: AGCCACATTTCATCATTCCCACTTC	NM_204794.3	100
FSHR	F: AATGGAACCTGCCTGGATGAGC R: CCCGATGGCTCCTTGGAAGAC	XM_040696841.2	87
LHR	F: CTCGTCCTCATAACCAGCCACTAC R: CACCGAAGCAATGAGCACCAAG	XM_010706641.2	111

**Table 3:** Effects of VE and KAE on production performance of late-laying hens.

Items	Groups				*p*-Value
	CON	VE	KAE	KAE + VE	
Egg laying rate (%)	89.56 ± 4.56 ^C^	91.50 ± 4.35 ^B^	91.89 ± 4.53 ^AB^	93.11 ± 3.45 ^A^	<0.001
Average daily feed intake (g)	120.55 ± 1.94	120.36 ± 1.60	120.29 ± 2.11	120.28 ± 1.51	0.817
Average daily egg weight (g)	57.12 ± 3.12 ^b^	58.22 ± 2.53 ^a^	58.48 ± 2.51 ^a^	58.73 ± 2.25 ^a^	0.025
Feed/egg ratio ^1^	2.14 ± 0.15 ^A^	2.09 ± 0.11 ^B^	2.06 ± 0.11 ^B^	2.06 ± 0.09 ^B^	0.004
Qualified egg rate (%)	99.59 ± 0.90	99.83 ± 0.73	99.80 ± 0.64	99.80 ± 0.65	0.192

Different lowercase letter superscripts mean notable differences (*p* < 0.05), while different capital letters show significant differences (*p* < 0.01). Values with the same or no letters mean no significant difference (*p* ≥ 0.05) ^1^ Feed/egg ratio = daily feed consumption/average egg weight. Data are presented as the mean ± SD.

**Table 4:** Effects of VE and KAE on egg quality of late-laying hens.

Items	Groups				*p*-Value
	CON	VE	KAE	KAE + VE	
Egg Shape Index	1.31 ± 0.05	1.32 ± 0.06	1.30 ± 0.05	1.32 ± 0.04	0.563
Haugh Unit	90.92 ± 4.84 ^b^	93.13 ± 4.73 ^ab^	94.46 ± 8.52 ^ab^	96.50 ± 7.89 ^a^	0.048
Eggshell Strength (kg/cm^2^)	3.68 ± 0.45 ^b^	3.95 ± 0.57 ^a^	4.03 ± 0.32 ^a^	4.10 ± 0.36 ^a^	0.017
Eggshell Relative Weight (g)	12.30 ± 1.08 ^b^	12.99 ± 0.90 ^a^	13.01 ± 0.95 ^a^	13.21 ± 1.13 ^a^	0.032
Eggshell Thickness (mm)	0.31 ± 0.03 ^b^	0.33 ± 0.03 ^a^	0.33 ± 0.02 ^a^	0.34 ± 0.02 ^a^	0.010
Yolk Color	6.48 ± 0.60 ^B^	6.93 ± 0.68 ^A^	7.12 ± 0.85 ^A^	7.18 ± 0.59 ^A^	0.002
Yolk Relative Weight (g)	25.54 ± 1.64 ^B^	26.84 ± 1.70 ^A^	27.09 ± 1.97 ^A^	27.11 ± 1.40 ^A^	0.008
Yolk-To-White Ratio	40.90 ± 4.13	41.92 ± 4.46	42.74 ± 5.02	44.85 ± 6.31	0.058

Different lowercase letter superscripts mean notable differences (*p* < 0.05), while different capital letters show significant differences (*p* < 0.01). Values with the same or no letters mean no significant difference (*p* ≥ 0.05). Data are presented as the mean ± SD.

**Table 5:** Effects of VE and KAE on the Alpha diversity of the cecal microbiota in late-laying hens.

Items	Groups				*p*-Value
	CON	VE	KAE	KAE + VE	
ACE	1157.00 ± 258.40	1044.00 ± 141.80	1133.00 ± 254.80	1083.00 ± 331.60	0.867
Chao1	1143.00 ± 266.60	1029.00 ± 133.40	1120.00 ± 256.90	1067.00 ± 321.20	0.927
Sobs	1062.00 ± 160.40	1013.00 ± 122.10	1058.00 ± 161.40	1004.00 ± 214.80	0.936
Shannon	5.53 ± 0.06	5.65 ± 0.15	5.64 ± 0.19	5.68 ± 0.25	0.531

Data are presented as the mean ± SD.

**Table 6:** Effects of VE and KAE on the relative abundance of species at the phylum level in the cecal microbiota in late-laying hens.

Items	Groups				*p*-Value
	CON	VE	KAE	KAE + VE	
Phylum level					
Firmicutes	42.35 ± 8.77	45.26 ± 9.38	46.72 ± 4.59	47.79 ± 11.15	0.892
Bacteroidota	45.34 ± 9.05	47.78 ± 11.17	43.79 ± 4.49	44.78 ± 8.82	0.941
WPS-2	3.40 ± 1.56	1.70 ± 1.15	2.474 ± 1.47	1.05 ± 0.85	0.119
F/B ^1^	0.96 ± 0.32	1.01 ± 0.37	1.03 ± 0.23	1.18 ± 0.54	0.858

^1^ F/B = Firmicutes/Bacteroides. Data are presented as the mean ± SD.

**Table 7:** Effects of VE and KAE on the Alpha diversity of the uterus microbiota in late-laying hens.

Items	Groups				*p*-Value
	CON	VE	KAE	KAE + VE	
ACE	659.90 ± 136.80	700.40 ± 167.50	764.70 ± 83.70	710.80 ± 21.95	0.644
Chao1	658.30 ± 136.50	700.00 ± 168.20	763.20 ± 84.10	709.80 ± 22.12	0.644
Sobs	656.30 ± 135.50	699.30 ± 169.40	755.70 + 84.76	709.00 ± 22.52	0.643
Shannon	4.77 ± 0.60	4.54 ± 0.76	4.67 ± 0.37	4.29 ± 0.16	0.668

Data are presented as the mean ± SD.

**Table 8:** Effects of VE and KAE on the relative abundance of species at the phylum level in the uterus microbiota in late-laying hens.

Items	Groups				*p*-Value
	CON	VE	KAE	KAE + VE	
Phylum level					
Firmicutes	57.56 ± 5.92	56.40 ± 14.30	62.78 ± 4.28	68.29 ± 11.67	0.442
Proteobacteria	23.86 ± 8.24	25.23 ± 20.29	18.84 ± 3.54	16.30 ± 6.339	0.764
Bacteroidota	8.917 ± 3.19	13.66 ± 10.75	11.16 ± 2.57	4.68 ± 2.785	0.340
F/B	4.46 ± 1.36 ^b^	4.51 ± 1.12 ^b^	6.27 ± 1.17 ^ab^	9.31 ± 3.36 ^a^	0.016

Different lowercase letter superscripts mean notable differences (*p* < 0.05). Values with the same or no letters mean no significant difference (*p* ≥ 0.05). Data are presented as the mean ± SD.

## Data Availability

Most of the data generated or analyzed in this study are presented in this published article. Additional data not included here are accessible upon reasonable request to the corresponding author.
